# Influence of Reduction with NaBH_4_ and HCl in Obtaining Amino Derivatives of Cashew Gum and Cytotoxic Profile

**DOI:** 10.3390/polym15132856

**Published:** 2023-06-28

**Authors:** Francisco das C. M. Brito, Wilton C. Lopes, Fábio O. S. Ribeiro, Raiza Raianne Luz Rodrigues, Klinger Antonio da França Rodrigues, Fabrício dos Santos Machado, Ana Jérsia Araújo, José Delano Barreto Marinho Filho, Antônia Carla J. Oliveira, Edson C. S. Filho, Irisvan S. Ribeiro, Regina C. M. de Paula, Fernando Hallwass, Vicente Gálber F. Viana, Durcilene A. Silva

**Affiliations:** 1 Federal Institute of Piauí, Campus, Piripiri 64260-000, PI, Brazil; 2 Biodiversity and Biotechnology Research Center, Biotec, Parnaíba Delta Federal University, UFDPar, Parnaíba 64202-020, PI, Brazil; wcarvalholopes@ifpi.edu.br (W.C.L.); fabioriber2014@gmail.com (F.O.S.R.); 3 Postgraduate Program in Materials Engineering PPGEM, Federal Institute of Piauí, Campus, Teresina 64000-040, PI, Brazil; galber@ifpi.edu.br; 4 Federal Institute of Piauí, Campus, Pedro II 64255-000, PI, Brazil; 5 Infectious Disease Laboratory, Ladic, Parnaíba, Delta Federal University, UFDPar, Parnaíba 64202-020, PI, Brazilklinger@ufpi.edu.br (K.A.d.F.R.); 6 Laboratório de Cultura de Células do Delta (LCCDelta), Parnaíba Delta Federal University, UFDPar, Parnaíba 64202-020, PI, Brazilanajersia@gmail.com (A.J.A.); delanomarinho@gmail.com (J.D.B.M.F.); 7 Interdisciplinary Laboratory for Advanced Materials, Teresina 64049-550, PI, Braziledsonfilho@ufpi.edu.br (E.C.S.F.); 8 Department of Organic and Inorganic Chemistry, Federal University of Ceará, Fortaleza 60440-900, CE, Brazil; 9 Department of Fundamental Chemistry, Federal University of Pernambuco, Recife 50670-901, PE, Brazil

**Keywords:** polysaccharide, cashew gum, modification, amin

## Abstract

Tree-exuded gums are natural polymers that represent an abundant raw material in the food and pharmaceutical industries. The cashew gum can be obtained by exudation of trees of the genus Anacardium, a native species of the Brazilian northeast; its polymer consists of monosaccharide units propitious to the action of chemical reactions that tend to improve their intrinsic characteristics among them, as the degree of hydro-solubility. The objective of this work was to modify the exudate gum of *Anacardium occidentale* (cashew gum (CG)) through an amine reaction. The modification was confirmed by Nuclear Magnetic Resonance (^1^H NMR), infrared spectroscopy (FTIR), gel permeation chromatography (GPC), zeta potential, and thermogravimetric analysis (TG). In addition, the chemical modification altered the molar mass and surface charge of the CG, and the amino group binding to the CG polymers was confirmed by FTIR spectra. In addition, cytotoxicity tests were performed where cell viability was estimated by an MTT assay on RAW 264.7 macrophages. Through these tests, it was found that the amine caused an increase in the thermal stability of the amino compounds and did not present cytotoxic potential at concentrations below 50.0 mg/L.

## 1. Introduction

Natural gums can be divided into vegetable exudates gums, plant seed gums, seaweed gums, and gums produced by microorganisms. The interest in gums obtained from plant exudates has grown considerably due to their structural properties and their functions in food, pharmaceutical, cosmetic, textile, and biomedical products [1].

Water-soluble gums, also known as hydrocolloids, are used for various applications such as dietary fibers, texture modifiers, gelling agents, thickeners, stabilizers, emulsifiers, coatings, films, encapsulating agents, and drying aids [2,3]. There is a strong tendency to replace synthetic materials with natural gums due to non-toxicity, low cost, safety, and availability [4]. The most industrially used gums are Starch, Cellulose, Guar gum, Arabic gum, Goethe, Korean, Tragacanth, Galena, and Agar. However, the search for new gums with special properties has aroused interest in the scientific community [5,6,7].

Cashew gum, an exudative gum, is a polysaccharide extracted from a low-cost and easily accessible source, the *Anacardium occidentale* tree, widely distributed in northeastern Brazil [8].

Some studies report the possibility of the use of cashew gum in several sectors, however, its applications in industrial environment are not well-established. Although cashew gum has been explored since 1970, it is only since the 1990s that it has received greater interest from the scientific community, mainly due to the structural and chemical similarity with gum Arabic. Gum Arabic, also known as Acacia gum, is considered to be the oldest and best known among natural gums; however, it has a high cost, and occasionally its import is compromised by the difficulty of supply due to climatic, economic, and political problems in the African producing region [9,10].

Cashew gum (GC) has similar characteristics to gum Arabic. It can be replaced as liquid glue for paper, in the pharmaceutical industry, in cosmetics, as a binder for capsules and tablets, and in the food industry as a stabilizer for juices, beers, and ice cream [11].

Studies have shown the positive activity of GC in wound healing [12], antimicrobial activity [13,14] and as a culture medium for bacteria [15], in hydrogel manufacturing, and for the treatment of effluents containing copper and zinc ions, as well as an anti-inflammatory agent in the healing process of mice [12]. Its differential is the high availability in the northeast region of Brazil, especially in the states of Ceará, Piauí, and Rio Grande do Norte. Gum extraction is a way of adding value to cashew tree culture [16].

Cashew gum (CG), an Arabinogalactan, is derived from the resin released by the tree species(*Anacardium occidental* L.). CG is mainly composed of a highly branched heteropolysaccharide structure, consisting of galactose in its main chain with β (1→3) connections and side chains with β connections (1→6). Arabinose is present as small branches or terminal groups. Glucose, rhamnose, mannose, and xylose halves are present as terminal groups [17,18]. Several studies have reported that GC has several bioactivities, such as antimicrobial [19,20], healing [12], encapsulation of larvicide [21], immunomodulating [6], antidiarrheal [7], and gastroprotective properties [22].

The cashew tree gum (CG), obtained from the exudate, which has a yellowish color and is released naturally or through incisions made in the trunk of the cashew tree (*Anacardium occidentale* L.), is a branched heteropolysaccharide. Chemical modifications in the structure of this polymer by introduction of new functional groups by using carboxymethylation [17], oxidation [18], sulphation [23], acetylation [8], and quaternization [14] have been carried out in order to obtain new physicochemical characteristics. However, no reactions of polymer functionalization have been carried out yet in order to make it cationic.

Cationic polysaccharides have wide applications in drug and gene delivery and in new areas such as biotronics applications and fluorescent labeling. Hence, to improve the usefulness of cashew gum, a cationic derivative was synthesized, and some of its properties are reported in this article. As one of the methods to improve the biological properties of polysaccharides, chemical modification has been widely used for commercial and specific interests. The incorporation of new functional groups by chemical modification causes structural changes of polysaccharides, consequently affecting their biological and physicochemical properties [24]. Among several chemical modifications, the amine produces the derivative that usually presents greater stability against hydrolysis and thermolysis and improved physical properties [25].Previously, beta glucans from cereal sources, such as oats, were chemically modified with amine, affecting biological activities, [26]. However, the amine of the cashew polysaccharide has never been reported to our best knowledge.

This versatile naturally occurring biopolymer has recently been used in the pharmaceutical industry [8,27] and food [20]. However, there is a lack of understanding of its physicochemical properties, thus limiting its use in food and pharmaceuticals. The chemical structure, solubility, and molecular weight of GC closely affect its solution properties as well as its interactions with other polysaccharides. GC modification can improve its technological and functional properties [20]. The long-term strategy to promote the use of GC in industry is therefore to understand and explore the physicochemical properties of gum, in its original or chemically modified state, isolated or mixed with other polymers.

Thus, in this study, cashew gum was modified by reductive amine reaction in two separate routes and with a different reducing agent for each route in order to identify the amino derivative with the highest degree of substitution. The derivatives were characterized, and the biological profile was tested in different cell lines.

## 2. Materials and Methods

### 2.1. Materiais

CG was obtained from the Cashew tree exudate (*Anacardium occidental* L.) The CG used in this work was from the municipality of Island Grand do Piauí located in the Piauiense Coastal Plain with coordinates 02°51′56″ S and 41°48′42″ W latitude and longitude to obtain the exudates produced by the species. Ethanol (C_2_H_6_O) was acquired from Dinâmica Química Contemporânea LTDA. (Indaiatuba, Brazil) Acetone (C_3_H_6_O), sodium borohydride (NaBH_4_), MTT 3-(4,5- dimethyl thiazole-2-yl)-2,5-diphenyl tetrazolium, and Ethylenediamine were acquired from Sigma-Aldrich.

The specimen *Anacardium occidentale* L. has already registered in the National System of Genetic Heritage Management and Associated Traditional Knowledge, with the number AAA024E.

### 2.2. Purification of the Cashew Gum

To obtain the gum, a method adapted from [28] was used. This used 10.0 g of crushed exudate immersed in 100.0 mL of deionized water, being under constant agitation for 12 h for the solubilization of the polymer. After solubilization, it was filtered in an analytical funnel for retention of impurities that may be adhered to the exudate. The pH was adjusted to 7 with NaOH at 0.1 mol.L^−1^ and 2.0 g of NaCl was added, leaving the solution in agitation for another hour. Subsequently, the polymer was recovered by precipitating it in ethanol at a 4:1 ratio (ethanol/gum). Sediment was expected, and filtration was carried out in a sintered plate funnel. The precipitate was washed two more times with 99.0% ethanol and once with acetone. The drying was performed with the aid of a mortar and pistil and dried under the continuous flow of hot air until obtaining powder.

### 2.3. Modification Reaction

**Figure 1:**
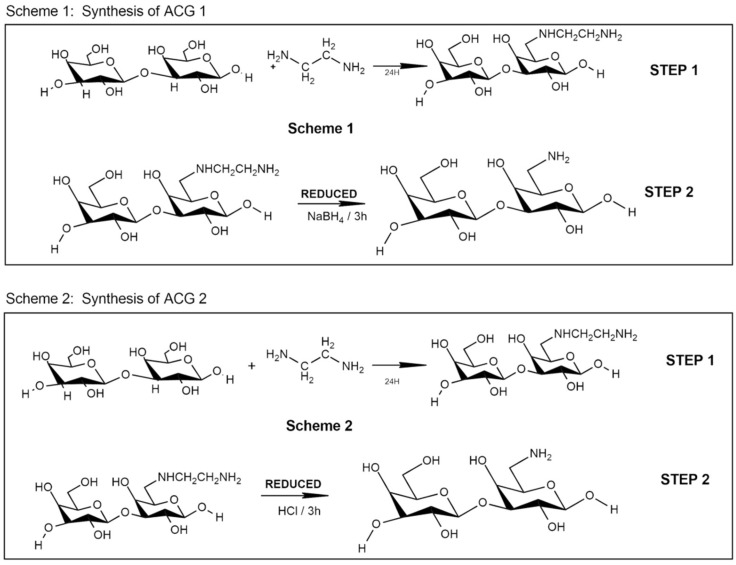
Mechanism of Cashew Gum Amination.

Two routes were used to obtain the modification reaction of Cashew Gum (CG) to compare their effectiveness. On route 1 it was performed and adapted as described by [22] by the addition of ethylenediamine (C_2_H_8_N_2_) as an amine agent by the substitution of the -OH group of the CG by -NHCH_2_CH_2_NH_2_ step 1. In addition, the -NHCH_2_CH_2_NH_2_ group was reduced to -NH_2_ by the addition of NaBH4 as shown in Figure 1 with [24]. In a 100.0 mL beaker (bottle 1), 1.0 g of CG was added and dissolved in 10.0 mL of distilled water, and stirred for 30 min to obtain a homogeneous solution. An amount of 12.0 mL of ethylenediamine was added, which was placed to react under agitation for 24 h at room temperature. Overtime, 5.0 mL of 5% NaBH_4_ solution was added to the reaction mixture contained in the flask. The CG modification route 2 (CG) was performed and adapted as described by [25], and using ethylenediamine (C_2_H_8_N_2_) as an amine agent by replacing the -OH group of the GC by -NHCH_2_CH_2_NH_2_ step 2 of Figure 1.

In a 100.0 mL beaker, 1.0 g of CG was added which was dissolved in 10.0 mL of distilled water, stirred for 30 min to obtain a homogeneous solution,12.0 mL of ethylenediamine. The homogeneous system was placed to react under agitation for 24 h at room temperature. After passing the time, we added 5.0 mL of HCl solution 1 mol.L^−1^, and the flask was subjected to vigorous agitation for a period of 3 h. After finishing the samples were precipitated, washed with 99% ethyl alcohol and acetone, and precipitated dried in a warm air stream, and then sprayed to uniform the particles.

After drying, a yield of 67% for ACG1 and 65% for ACG2 was obtained.

### 2.4. Characterization of Cashew Gum Amine

#### 2.4.1. Fourier Transform Infrared Spectroscopy (FTIR)

The infrared spectroscopy was performed in an infrared spectrometer by Fourier transform (Nicolet iS5-iD7 ATR-Thermo Fisher Scientific Mississauga, Mississauga, ON, Canada) using the technique of total attenuated reflection, with a spectrum ranging from 4500 to 400 cm^−1^.

#### 2.4.2. Thermogravimetric Analysis (TGA)

The thermograms were obtained through the thermogravimetric analyzer (TGA51 Thermogravimetric Analyzer SHIMADZU), in a nitrogen atmosphere with a flow of 50 mL/min, heating rate of 10 °C/min, and range of analysis from 25° to 600 °C. The sample was packed at platinum sample ports and a mass of 4.0 0.1 mg.

#### 2.4.3. Elemental Analysis(C, H, N)

For the determination of the percentage of carbon, nitrogen and hydrogen, the samples were processed in duplicates in an Elemental Analyzer-Perkin Elmer model PE 2400 in CHNS mode with thermal conductivity detector.

#### 2.4.4. Degree of Substitution

The degree of substitution (*DS*) of the CG derivatives was determined based on that of the % *N* (nitrogen percentage) obtained from the elemental analysis (minus the insignificant amount present in CG), and calculated according to the following Equation(1) [29]. *DS* was defined as the number of hydroxyl groups replaced per unit of sugar CG.

(1) $$DS=\frac{162\times \left(\frac{N\;\left(\%\right)}{14}\right)}{100-\left[\left(\frac{13}{14}\times N\left(\%\right)\right)\right]}$$

#### 2.4.5. Zeta Potential

Zeta potential measurements were analyzed in a Malvern Zetasizer Nano Modelo ZS90 for each sample at a concentration of 1.0 mg.mL^−1^. The Zeta potential (mV) was determined from the measurements the samples were diluted in Milli-Q water (1:10; *v*/*v*)as suggested by [30]. The parameters were estimated by the average of three measures.

#### 2.4.6. Gel Permeation Chromatography (GPC)

The molar mass distribution was determined by gel permeation chromatography (GPC) in the Shimadzu LC-20AD equipment coupled to a refractive index detector (RID-10A). For the analysis, a column PolySep-GFC-P Linear (300 × 7.8 mm) and NaNO_3_ (a) (0.1 mmol·L^−1^) were used as eluent. The measurement was made at 30 °C, with a flow of 1.0 mL/min, and the injected volume of the sample was 50.0 µL. Molar mass was calculated using a pullulan curve (Equation (2)) or (Equation (3)).

(2) Log Mpk=14.408442−1.157087×Ve

(3) Log Mpk=14.285228−1.167733×1.167733×Ve

Note: Equation (2) was used only for the samples GCA1 and GCA2.

#### 2.4.7. Nuclear Magnetic Resonance Spectroscopy (^1^H NMR)

To obtain the NMR spectra, a sample of CG and ACG (20.0 mg) each was dissolved in deuterium oxide (D_2_O) ^1^H spectra obtained from the NMR spectrometer Agilent 400 MHz at 40 °C.

#### 2.4.8. Cytotoxicity

##### Cell Lines and Cell Culture

The human and murine cell lines used in this work were kindly provided by the National Cancer Institute (Bethesda, MD, USA). The cell lines were cultured in cell culture flasks (25 cm^2^ or 75 cm^2^ volume) in supplemented DMEM medium (10% SFB, 1% antibiotic −100 U/mL penicillin and 100 μg/mL streptomycin) (Sigma-Aldrich, St. Louis, MO, USA), pH 7, at 37 °C, 5% CO_2_ with 80% humidity. The cell lines used were MDA-MB-231 (human breast adenocarcinoma), HCT-116 (colorectal carcinoma), L929 (mouse fibroblast), and RAW 264.7 (murine macrophages).

##### MTT Assay

The cells were plated into a 96-well multiplate at a density according to the doubling time of each lineage (8 × 10^4^ cells/mL of MDA-MB-231; 6 × 10^4^ cells/mL of the HCT-116; 10 × 10^4^ of L929; 10 × 10^4^ cells/mL of RAW macrophages 264.7). After 4 h of incubation at 37 °C and 5% CO_2_ for cell adhesion, three washes with sterile PBS were performed. Then, 100.0 μL of supplemented DMEM medium containing different concentrations of substances (50.0 to 1.56 mg/mL) was added and incubated for a period of 72 h. At the end of the period 10.0 μL of MTT (5.0 mg/mL) was applied and incubated for more than 4 h. Then the supernatant was removed and 100.0 μL of DMSO was added in all wells. After 30 min of agitation, the reading was performed at 540 nm in a plate reader. Supplemented DMEM medium containing 0.5% DMSO was used as negative control and considered as 100% macrophage viability.

## 3. Results

### 3.1. Fourier Transform Infrared Spectroscopy

The chemical characterization was performed to confirm the chemical modification of cashew gum by the insertion of amino on the polysaccharide surface. In the spectra, it is possible to observe characteristic bands of O-H (3290 cm^−1^) and the emergence of a shoulder (3345 cm^−1^) corresponding to the replacement of OH groups by NH_2_ groups on the surface of CG [8].We can also identify bands at 1132 cm^−1^, 1078 cm^−1^,and 1013 cm^−1^ attributed to C-O-C angular deformations of glycosidic bonds and O-H bending of alcohols and that are also present in ACG2.

The most striking difference between CG and its amine derivatives was the new absorption bands at 1250–1300 cm^−1^ and 700–800 cm^−1^.It is possible to observe a weak band at 1572 cm^−1^, which corresponds to the angular deformation of NH_2_ and the band at 1456 cm^−1^ due to angular deformation of primary amine N-H bond confirming the chemical modification of CG [31,32]. Bands are observed in the regions of 1366 cm^−1^ and 1323 cm^−1^ referring to the angular deformation of germinal CH_3_ appearing as doublet and evident only in ACG1 [17].

**Figure 2:**
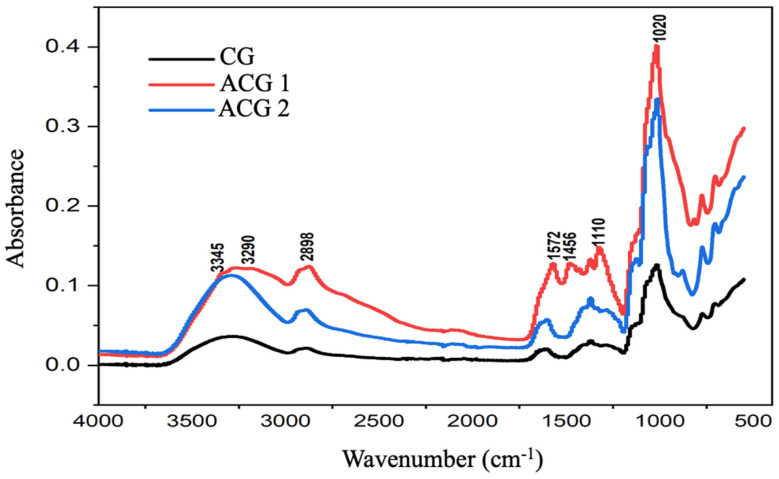
Spectrum of ftir CG, CGA1 and CGA2.

The ACG2 sample presented a spectrum similar to that of the CG with some displacements, with no significant changes and/or emergence of new bands as shown in Figure 2.

Changes in the chemical structure of polysaccharide gums can lead not only to changes, but also to adding new properties. Modification of the amine of polysaccharide gums can create amphiphilicity properties by integrating the alkyl chains of the amine or creating a positive charge after the introduction of a primary amine into the structure.

### 3.2. Thermogravimetric Analysis (TG)

**Figure 3:**
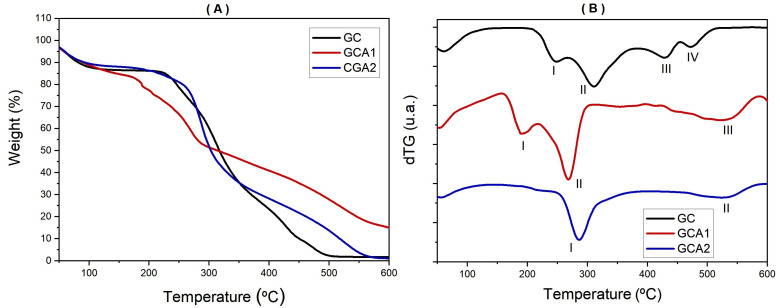
Thermogravimetric curves Tg (**A**) and dTG (**B**) for cashew gum and amine derivatives in N_2_ atmosphere at a heating rate of 10 °C/min. (I, II, III and IV temperatures at which events occur).

The thermogravimetric curves of cashew gum and amine derivatives show weight loss events in four, three, and two stages for GC, CGA1, and GCA2, respectively, shown in Figure 3 and the parameters obtained from the curves in Table 1. The initial decomposition temperature (To) is above 200 °C, as verified for some other polysaccharides [33]. The comparison of the curves reveals that the thermal decomposition processes of the samples are different and that the first event occurs at (Tmax. = 60 °C) related to water loss and is present in all curves.

The CGA1 and CGA2 gums show a new event at Tmax = 531 °C that is not present in the original gum TG. Similar results were obtained for carboxymethylated cashew gum [34]. This event is probably related to the thermal fission of carbohydrate segments.

However, the Tmax of the second event (309 °C) is similar to that previously reported for cashew gum [35]. The maximum degradation temperature in the range of 270–320 °C was determined for other polysaccharides: gum Arabic (316 °C) [33] and chitosan (300 °C) [36]. The decomposition of derivatives of some polysaccharides, such as guar gum [37] and galactoxyloglucan [38], also begins at a lower temperature than that of the original polymer. Below 200 °C, the amine derivatives had less weight loss than GC. Subsequently, weight loss increases sharply with temperature. However, above 330 °C, ACG1 and ACG2 had a lower weight loss than CG, an important factor regarding the thermal stability of CG after the amine reaction, corroborating with the results found by [24].

### 3.3. Elementary Analysis (C, H, N)

The elemental analysis of CG and ACG samples indicated the presence of 36.03% and 32.12% carbon, 5.68% and 6.01% hydrogen, 0.76% and 8.73% nitrogen, respectively to CG and ACG2 (Table 2). The increase in the percentage of nitrogen in the product confirms that the amino group was incorporated into the polymer skeleton. The results clearly indicate the presence of the NH_2_ group on both routes and confirm the modification of the CG. The small amount of nitrogen in CG reflects the presence of protein traces (0.15%). The ACG1 (route with NaBH_4_) presented a greater DS compared to that observed fromACG2 (route with HCl). Therefore, the process of route 1 was more effective than route 2; it is expected that the amended results can create ionic imbalances in the biopolymer due to the formation of NH_2_ [26].

### 3.4. Zeta Potential

The surface charge is an important parameter to be investigated in the characterization of modified gums because this factor can influence its properties involving electrostatic interactions. The zeta potential measurement reflects the effective charge of the particle and refers to the electrostatic repulsion between them. The results indicate that the CG-modification process promoted a change in the polymer surface charge. The CG surface charge is −26.5 mV, and this negative value is justified by the presence of glucuronic acids (13.5%) in the CG composition. After modification, lower anionic characters of −3.6 mV for ACG2 and +0.16 mV for ACG1 were observed, probably due to the insertion of positively loaded amino groups, previously proven by FTIR, in the CG polymeric chain.

### 3.5. Gel Permeation Chromatography

**Figure 4:**
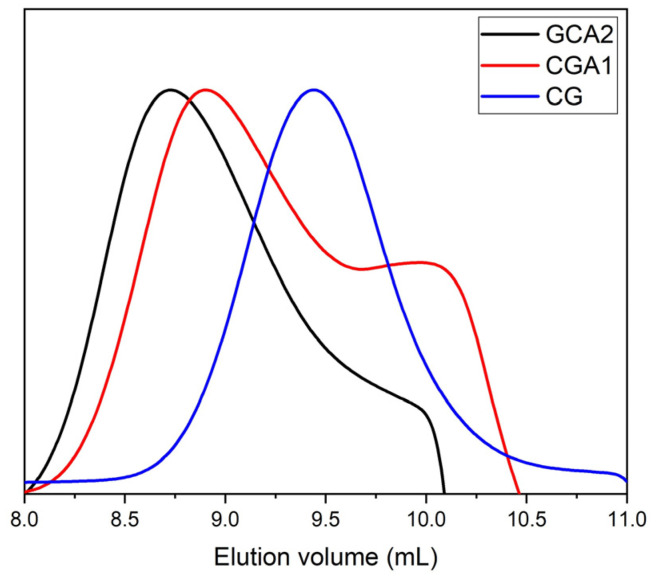
Mass distribution in the GPC.

Natural gums are composed of polysaccharides of multiple sugar units linked together to form large molecules; they have a high molecular weight. However, chemical modification can cause changes in the molecular weight of CG, which can influence physicochemical properties of CG. Thus, the GPC analysis was performed to investigate possible changes in the molecular mass of the CG after chemical modification by amination. The amines and the original gum Figure 4 and (Table 2) presents physicochemical and mass distribution data, where we can conclude that they are polydispersed macromolecules, with polydispersity values (Mw/Mn) of 6.1 and 3.6 for ACG1 and ACG2, respectively. We can still observe that the molecules of ACG1 and ACG2 present bimodal distribution indicating the formation of amino products similar results found in [17] These results corroborate with the results obtained by [24], who performed this same synthesis pathway to obtain the xyloglucan gum amine, where this reaction also promoted reduction in the molecular mass of the modified polysaccharide.

### 3.6. Hydrogen Magnetic Resonance (NMR ^1^H)

**Figure 5:**
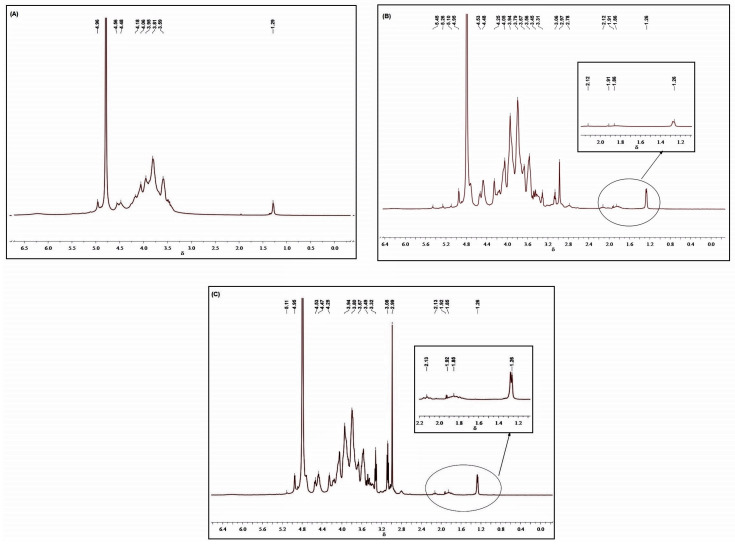
NMR spectra CG(**A**), ACG1 (**B**), and ACG2 (**C**).

Figure 5 Shows the ^1^H NMR spectra of CG(A), ACG1(B), and ACG2(C), respectively. The NMR spectrum for cashew gum without modification in Figure 5A showed characteristic anomeric protons in the region of 4.4 to 5.0 ppm using D_2_O as solvent [23]. Signals in this region are reported as a duet α-D-glucose (4.95 ppm), α-L-rhamnose (4.81 ppm), β-D-galactose (1→3) (4.69 ppm and 4.43 ppm), and glycuronic acid (4.51 ppm). The H-2 to H-5 Figure 5B,C signals are superimposed in the regions of 3.4 ppm to 4.3 ppm, and a quartet signal in the region of 1.26 ppm is due to the methyl protons of rhamnose [39]. Signals in the region of 1.5 to 2 ppm are due to protons of NH_2_ (Figure 5B,C). Comparing to native GC (Figure 5A), ACG1, and ACG 2 (Figure 5B,C, respectively) exhibited an additional peak close to 3.1 ppm, which was attributed to methyl groups attached to primary amines [40]. The existence of this signal is a result of the hydrogen atom of the substituent that was introduced into the chain. In Figure 5C it is more intense, and thus, we can conclude based on the results of the zeta potential and elemental analysis that the sample ACG 2 presents a lower degree of substitution.

### 3.7. Cytotoxicity

Cytotoxicity analysis is a prerequisite for assessing the biocompatibility of biomaterials. In this context, cell viability was estimated by the MTT assay on RAW 264.7 macrophages, and the results are expressed as the percentage of the sample group in relation to the blank control. The results demonstrated a CC50 of 48.3 (mg/mL) for ACG1 and 40.02 (mg/mL) for ACG2. These results point to low cytotoxicity in RAW 264.7 macrophages at concentrations up to 25.0 (mg/mL), which showed viability above 65% for both ACG1 and ACG2 samples, indicating their good cell compatibility. In contrast, the highest tested concentration of 50.0 (mg/mL) showed a decrease in viability. These results corroborate those found by [41] who report low cytotoxicity for RAW 264.7 macrophages with polysaccharides. Our results indicate that the tested samples do not present cytotoxic effects at concentrations up to 25.0 (mg/mL) for this immunological cell line, and are, therefore, a valid and safe alternative for biomedical applications.

**Figure 6:**
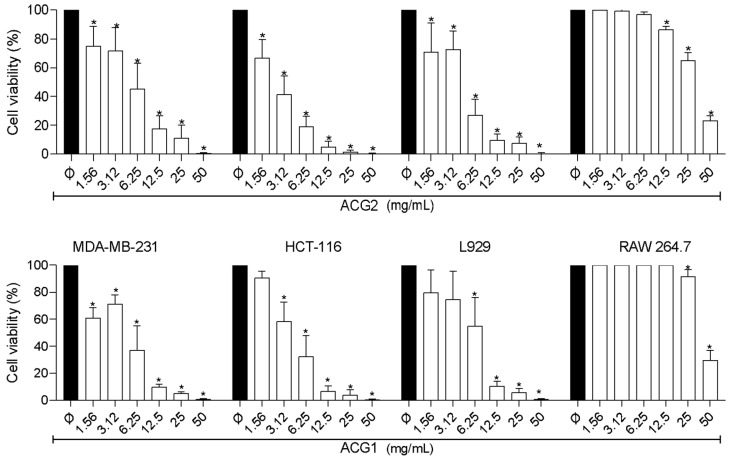
Cytotoxicity on macrophages RAW 264.7 and evaluation of the cytotoxic activity in vitro of modified cashew gum against HCT-116, MDA-MB-231, and L929 strains, after 72 h of incubation determined by the MTT method. ((*) concentration of the amine derivative used in the test).

Cytotoxicity analysis was also performed on mouse fibroblast cell lines (L929), human colorectal carcinoma (HCT-116), and human mammary adenocarcinoma (MDA-MB-231). Figure 6, together with the IC50 values shown in Table 3, shows the low selectivity of the molecules tested, with no significant differences between tumor and non-tumor cells. The cytotoxicity profile of the cashew tree gum modifications in a 72 h treatment shows that ACG1 and ACG2 showed a significant reduction (*p* < 0.05) compared to the control, for all strains tested, especially at the highest concentrations (Figure 5). These data indicate that the effect of cytotoxicity was dose-dependent, so that, with the increase in its concentration, cell viability was reduced.

Some polysaccharides may have in vitro and in vivo biological activities against tumor cells, but most of them have only a considerable effect at high doses. Previous work using cashew gum against cancerous and non-cancerous strains did not observe a cytotoxic effect, and this is possibly due to the low concentrations used [42], for example, did not observe cytotoxicity when using concentrations < 0.1 (mg/mL) in the HCT-116 strain, in their work on the antitumor potential of cashew tree gum. In their study, [43] managed to observe a moderate effect of this same strain, with an IC50 value > 0.5 (mg/mL) in their study of the cytotoxic profile of cashew tree gum. By using concentrations between 0.16 and 2.5 (mg/mL) of a polysaccharide obtained from Juniperus convallium, [41] found a significant antiproliferative effect on the cell lines used in their study, including the MDA-MB-231 line age.

The IC50 values are presented in mg/meal and were calculated from nonlinear regression using GraphPad Prism Software version 8.0. CI50da Doxorubicin (positive control) at HCT-116: 0.12 µg/mL 0.09–0.17); MDA-MB-23: 0.31 µg/mL (0.22–0.39); L929: 0.42 µg/mL (0.30–0.62).

## 4. Conclusions

In this study, the amination of cashew gum was performed using an easy and simple synthetic strategy. The modification of the polysaccharide improved the thermal properties of the CG; it is observed that the route 1 is more effective in the amination synthesis of the cashew gum.

Modified gums showed low invitro cytotoxicity at concentrations lower than 50 mg/L. The invivo cytotoxicity profile of cashew gum modifications in a 72 h treatment shows that ACG1 and ACG2 showed a significant reduction (*p* < 0.05) control, for all strains tested, especially in the highest concentrations. These data indicate that the effect of cytotoxicity was dose-dependent, so that, with the increase inits concentration, cell viability was reduced. These properties can be attractive for safe applications to biomedicine.

## Figures and Tables

**Table 1:** Thermal analysis data of cashew gum and it derivates in N2.

Data	CG	CGA1	CGA2
Tinitial ^a^ (°C)	247	192	215
Tinitial ^b^ (°C)	473	531	531
Tmáx (°C) in the range			
220–260	248 (sh)	189 (I)	-
260–350	310 (II)	270 (II)	287 (I)
400–450	428 (III)	-	-
450–600	473 (IV)	532 (III)	530 (II)
Residual mass 800 °C (%)	1.7	1.7	1.7

Sh, shouder. ^a^ Initial decomposition temperature (TGA curve on set). ^b^ Final decomposition temperature.

**Table 2:** Data of physical and chemical properties of gums and derivatives.

	CG		CGA1		CGA2	
Moisture	6.7		5.6		5.6	
Ashes	1.7		1.8		1.1	
pH	7.43		7.53		7.50	
Zeta	−26.5		+0.16		−3.68	
Elementary analysis	%C	36.06	%C	32.12	%C	36.29
	%H	5.68	%H	6.01	%H	5.24
	%N	0.76	%N	8.73	%N	2.74
Protein	0.15		1.72		0.54	
DS	-		1.09		0.32	
Mpk (g/mol)	2.29 × 10^4^		7.83 × 10^3^		1.25 ×10^4^	
Mn (g/mol)	4.00 × 10^3^		1.07 ×10^3^		3.04 ×10^3^	
MW (g/mol)	2.12 × 10^4^		6.56 × 10^4^		1.10 × 10^4^	
IPD	5.3		6.1		3.6	

**Table 3:** Evaluation of the cytotoxic activity in vitro of modified cashew gum against HCT-116, MDA-MB-231 and L929 strains, after 72 h of incubation determined by the MTT method.

Sample	IC_50_ (mg/mL) Confidence Interval 95%			
	MDA-MB-231	HCT-116	L929	RAW 264.7
ACG1	3.2 (2.5–3.9)	4.1 (3.5–4.6)	6.9 (6.0–7.9)	>50
ACG2	4.0 (3.2–5.0)	2.4 (2.2–2.6)	3.9 (3.3–4.5)	>50

## Data Availability

The data presented in this study are available only by the requesting them from the corresponding author due to the further work in progress.
